# Tunable Bound States in the Continuum in All-Dielectric Terahertz Metasurfaces

**DOI:** 10.3390/nano10040623

**Published:** 2020-03-27

**Authors:** Xu Chen, Wenhui Fan

**Affiliations:** 1 State Key Laboratory of Transient Optics and Photonics, Xi’an Institute of Optics and Precision Mechanics, Chinese Academy of Sciences, Xi’an 710119, China; chenxu@opt.ac.cn; 2 Center of Materials Science and Optoelectronics Engineering, University of Chinese Academy of Sciences, Beijing 100049, China; 3 Collaborative Innovation Center of Extreme Optics, Shanxi University, Taiyuan 030006, China

**Keywords:** terahertz, dielectric metasurfaces, graphene, quasi-bound states in the continuum, tunable

## Abstract

In this paper, a tunable terahertz dielectric metasurfaces consisting of split gap bars in the unit cell is proposed and theoretically demonstrated, where the sharp high-quality Fano resonance can be achieved through excitation of quasi-bound states in the continuum (quasi-BIC) by breaking in-plane symmetry of the unit cell structure. With the structural asymmetry parameter decreasing and vanishing, the calculated eigenmodes spectra demonstrate the resonance changes from Fano to symmetry-protected BIC mode, and the radiative quality factors obey the inverse square law. Moreover, combining with graphene monolayer and strontium titanate materials, the quasi-BIC Fano resonance can be tuned independently, where the resonance amplitude can be tuned by adjusting the Fermi level of graphene and the resonance frequency can be tuned by controlling the temperature of strontium titanate materials. The proposed structure has numerous potential applications on tunable devices including modulators, switches, and sensors.

## 1. Introduction

Terahertz (THz) waves have attracted significant interest in their promising applications in chemical identification, security screening, and sensing [1,2]. However, the development of THz technology has been limited due to the lack of efficient materials and devices [3]. Metamaterials have provided a promising way to solve this problem with their unique ability for manipulating THz waves. As the two-dimensional analog of metamaterials, metasurfaces respond to incident radiation mainly through their geometrical patterns, providing a new way to control THz waves and develop efficient THz devices [4,5]. For conventional metallic metasurfaces, due to the inevitable disadvantages such as Ohmic losses, large dispersive refractive index, and radiation losses from *LC* resonances [6,7], the performance and efficiency of metallic metasurfaces devices are limited [8]. Fortunately, as a promising alternative to metallic metasurfaces, all-dielectric metasurfaces can support the Mie resonances (electric dipole, magnetic dipole, and other multipolar resonances) with small dissipation and low thermal conductivity [9], thus providing a good platform for high-efficiency THz devices [10,11,12]. Moreover, all-dielectric metasurfaces are more favorable to realize flexible phase control because their thicknesses are relatively larger than that of metallic metasurfaces [13]. Recently, all-dielectric metasurfaces have received special attention and been widely used for wavefront manipulation, biomedical imaging, communication, and spectroscopic characterization [13,14,15]. More importantly, metasurfaces with high-quality factors (*Q*-factors) provide an efficient platform for ultrasensitive sensors, perfect absorbers, and nonlinear optics with strong localization of electromagnetic energy, which is the research topic in the field of dielectric metasurfaces [16,17,18]. With the radiation losses suppressed, Fano resonance provides an approach to achieve the high *Q*-factor resonance and has attracted lots of research interest due to the asymmetric lineshape and sharp spectral profile [19]. Generally, Fano resonance arises from the destructive interference between a bright (continuum) mode and a dark (discrete) mode [7]. The bright mode is superradiant and appears as a broad dipole resonance in the spectrum. The dark mode is subradiant and can only be excited indirectly through near-field coupling with the bright mode, which in turn interferes with the bright mode to produce Fano resonance. Numerous designs of the unit cell in the Fano system have been proposed, such as single-particle, dual-particle, and multi-particle systems [20]. In addition, one particularly effective approach to achieve extremely high *Q* Fano resonances is based on the bound states in the continuum (BIC) [21], where the disappearance of the Fano feature and the *Q*-factor going infinity due to the resonance uncoupled to the free-space radiation [22]. BIC was originally proposed by Friedrich and Wintgen in quantum mechanics, and then extended to acoustics, hydrodynamics, and optics [23]. Practically, BIC can be realized as quasi-BIC by introducing the structural asymmetry in the unit cell structure, where both the *Q*-factor and the resonance linewidth become finite [24]. It was revealed that dielectric metasurfaces with broken in-plane symmetry of unit cells can support high *Q*-factor resonance arising from the distortion of symmetry-protected BIC [25]. Such a BIC-inspired mechanism allows a general strategy to access extremely high *Q* resonances and giant enhancement of electromagnetic fields [26], realizing many useful functionalities including lasing and biosensing [27,28].

Generally, most of the aforementioned dielectric metasurfaces exhibit fixed functionalities once fabricated, which is unable to support a dynamic resonance and thus limit many practical applications. By integrating with active materials such as semiconductors, liquid crystals, graphene, and phase-change materials [21,29,30,31,32,33], some metasurfaces were proposed with tunable characteristics whereas only the resonance amplitude or frequency can be tuned. For example, the optically induced dynamic control and modulation of sharp BIC resonances are demonstrated by Fan et al. and Han et al. in the silicon-based THz metasurfaces [21,29]. However, all-dielectric quasi-BIC metasurfaces with independently tunable resonance amplitude and frequency have rarely been reported, which increases their applicability for multidimensional manipulation of THz waves. Since the surface conductivity can be tuned via shifting Fermi level under external bias voltage, graphene is commonly used in active THz metamaterials devices [34,35,36] and it is a good method to incorporate graphene with dielectric metasurfaces for active tunability [37]. Furthermore, phase-change materials are capable of providing great variations in material properties during the phase transition and can be used to design dynamic THz devices [38,39]. As one of the most popular ferroelectric phase-change materials, strontium titanate (SrTiO_3_, STO), with a strong ferroelectric soft mode, its dielectric behavior in the THz range can be fully controlled by external temperatures [11,40,41].

In this paper, inspired by the dynamic characteristics of graphene and STO materials, a tunable BIC-inspired THz dielectric metasurface combined with graphene monolayer and STO is proposed, where its amplitude and resonance frequency can be tuned independently. By shifting the Fermi level of graphene, the resonance amplitude exhibits a distinct modulation and the resonance frequency can be tuned by changing the temperature of the STO film. To the best of our knowledge, this is the first time to study the tunable quasi-BIC Fano resonance metasurface with multidimensional and independently manipulation of resonance amplitude and frequency of THz waves, offering great prospects for designing tunable THz devices.

## 2. Structure and Methods

**Figure 1:**
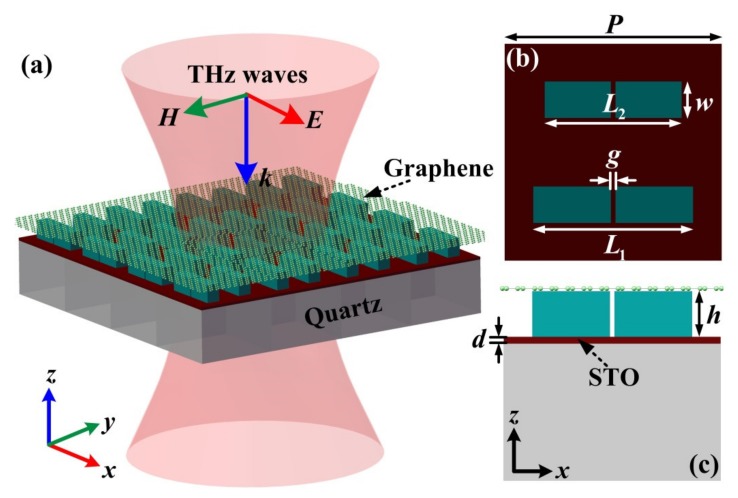
(**a**) Schematic view of the proposed metasurface structure, which is integrated with the strontium titanate (STO) film and the graphene monolayer. (**b**,**c**) Top and *y–z* cross-section view and geometric parameters of the unit cell structure.

Figure 1a shows the schematic diagram of the proposed structure, which consists of graphene monolayer, STO film, and dielectric metasurfaces. The unit cell structure in Figure 1b is composed of two rectangular bars with middle split gaps and different lengths *L*_1_ and *L*_2_. So, the in-plane asymmetry of the unit cell is controlled by bar lengths, and the asymmetry parameter can be defined as *α* = (*L*_1_ − *L*_2_)/*L*_1_. LiTaO_3_ is chosen as the dielectric bar which exhibits a strong polaritonic response and can be realized through crystal growth [42]. The complex permittivity of LiTaO_3_ can be expressed as:

(1) $$\varepsilon =\varepsilon_{\infty }\frac{\omega^{2}-\omega_{L}^{2}+i\omega \gamma }{\omega^{2}-\omega_{T}^{2}+i\omega \gamma }$$

where the transverse optical phonons frequency is *ω_T_*/2*π* = 26.7 THz, and the longitudinal optical phonons frequency is *ω_L_*/2*π* = 46.9 THz, *γ*/2*π* = 0.94 THz is the damping factor, and *ε*_∞_ = 13.4 is the limiting value with frequency much higher than *ω_L_*. The permittivity of LiTaO_3_ can be set as 41.4 with negligible dissipation losses with a frequency lower than phonon resonance [42]. The geometric parameters of the unit cell are: period *P* along *x*- and *y*-directions is set as 190 μm, the middle gap *g* is 4 μm, two bars with lengths of *L*_1_ = 140 μm, *L*_2_ = 120 μm and with same width and height of *w* = 30 μm, *h* = 44 μm. Figure 1c shows the *y–z* cross-section view of the unit cell, where the structure sequentially from bottom to top comprises quartz substrate, STO film, dielectric metasurfaces, and graphene monolayer, respectively. The thickness of the quartz substrate and STO film are, respectively, 500 μm and 0.4 μm. The graphene monolayer can be electrically modeled as an infinitesimally thin conductive layer characterized by a complex surface conductivity that is related to the Fermi level. The surface conductivity of graphene monolayer can be expressed as *σ_g_* = *σ_intra_* + *σ_inter_*, with *σ_intra_* and *σ_inter_* being the intraband transition and the interband transition, respectively. In the THz regime, the contribution of interband transition can be negligible compared with the intraband transition, and the intraband conductivity can be modeled by Drude-like expression as [35]:

(2) $$\sigma_{\mathrm{int}\mathrm{ra}}=\frac{ie^{2}E_{f}}{\pi {ћ}^{2}(\omega +i\tau^{-1})}$$

where *ћ* is the reduced Planck constant, *E_f_* is the Fermi level of graphene, and *τ* = *μE_f_*/(*ev_f_*^2^) is the carrier relaxation time. Fermi velocity *v_f_* is set as 10^6^ m/s, and the carrier mobility *μ* is chosen as 3000 cm^2^/Vs, much smaller than theoretical values and falls within the range of experimental capacity [43]. Thus, the permittivity of graphene can be calculated as *ε_g_* = 1 + *iσ_intra_*/(*ε*_0_*ωt_g_*), where *t_g_* = 1 nm is the thickness of graphene used in the simulation. Generally, by controlling the temperature, ferroelectric materials are suitable for modulating the permittivity, which is characterized by the existence of a strong polar soft lattice vibrational mode responsible for the ferroelectric phase transition [33]. The contribution of the soft mode to the low-frequency dielectric permittivity is very high and the dynamics of the soft mode allows tuning of the dielectric permittivity because the mode frequency decreases upon cooling [44]. Therefore, the ferroelectric materials STO exhibit a big potential for high dielectric tunability with reasonably low losses in the THz regime by means of temperature. Much work has been reported for the temperature-dependent dielectric properties of STO materials [33,40,41], where the complex permittivity can be expressed as [44]:

(3) $$\varepsilon_{\omega }=\varepsilon_{\infty }+\frac{f}{\omega_{0}^{2}-\omega^{2}-i\omega \gamma }$$

where *ε*_∞_ ≈ 9.6 is the high-frequency bulk permittivity, the temperature-independent oscillator strength *f* is 2.3 × 10^6^ cm^−2^, and *ω* is the angular frequency. *ω*_0_ and *γ* are the soft mode frequency and damping factor, calculated as:

(4) $$\omega_{0}\left(T\right)\left[{\mathrm{cm}}^{-1}\right]=\sqrt{31.2\left(T-42.5\right)}$$

(5) $$\gamma \left(T\right)\left[{\mathrm{cm}}^{-1}\right]=-3.3+0.094T$$

Due to softening of the ferroelectric soft mode, the complex permittivity of STO increases considerably upon cooling. Hence, STO has temperature-dependent permittivity and can be used to construct tunable metasurfaces. In addition, separated by the middle dielectric metasurface array, the effect of varying the temperature of the STO film on the graphene monolayer can be ignored. To investigate the proposed structure, the calculations with the frequency domain solver and tetrahedral mesh type were performed by electromagnetic full-wave simulation CST microwave studio. The unit cell boundary conditions were applied in *x*- and *y*-directions to characterize the periodic structure, and the open boundary condition was employed along the *z*-direction in the free space. The THz wave was a normal incidence with the electric field along the *x*-axis.

## 3. Results and Discussions

**Figure 2:**
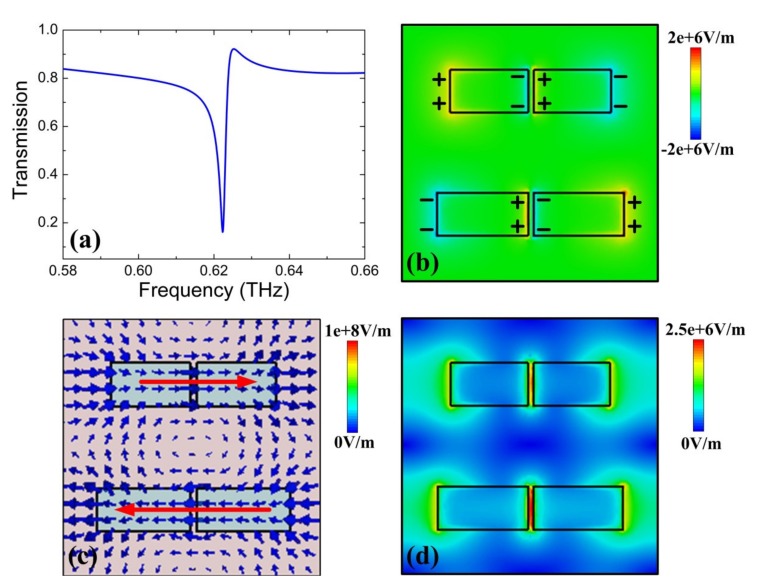
(**a**) Transmission spectrum of the proposed metasurface structure. Distributions of (**b**) electric field *E_z_* (**c**) displacement currents and (**d**) electric field amplitude |*E_z_*| at resonance dip. The red arrows indicate directions of displacement currents.

Generally, for metallic metasurfaces, the electromagnetic fields are mainly confined around the metal interface. Nevertheless, for dielectric metasurfaces, they are concentrated within the dielectric, which limits the light interaction with the surrounding medium [19]. Therefore, the split gaps are introduced in dielectric bars of the unit cell to improve the localized electromagnetic fields and the light interaction with the surrounding medium, due to the slot waveguide effect [45]. At first, the transmission spectrum of the proposed structure is investigated, with the length difference Δ*L* = 20 μm, the Fermi level of graphene monolayer set as 0 eV, and the temperature of the STO film set as 300 K. In Figure 2a, one can observe a sharp resonance dip at 0.6223 THz and the transmission profile with a characteristic of dip/peak pair has a distinct asymmetric Fano lineshape [20]. By fitting the transmission spectrum with a typical Fano formula given by *T_Fano_* = |*a*_1_ + *ia*_2_ + *b*/(*ω* − *ω*_0_ + *iγ*)|^2^, where *a*_1_, *a*_2_, and *b* are real constant numbers, *ω*_0_ is the resonance frequency, and *γ* is the damping rate, the *Q*-factor can be calculated as 212.39 with *Q* = *ω*_0_/2*γ* [20], which is a high *Q* value in THz metasurfaces. Moreover, to qualitatively analyze the underlying physics of the Fano resonance, the distribution of the *z*-component electric field *E_z_* is simulated, as shown in Figure 2b. One can clearly observe the electric field *E_z_* with strong enhancement occurring at both ends and middle gaps in the dielectric bars. The charge distribution at the resonance frequency shows an opposite in these two bars (shown by positive and negative charges), which is an electric quadrupole resonance. Moreover, in Figure 2c, the displacement currents at the resonance frequency are also simulated. Clearly, the anti-phased oscillation displacement currents and the opposite direction (shown by red arrows) can be induced, in which these two opposite displacement currents interfere destructively, thereby generating the high *Q* Fano resonance. In addition, a lot of opposite charges have been confined at two sides of the gaps in Figure 2b, leading to strong electric field enhancement and electromagnetic energy localized at the gaps, as shown in Figure 2d. Therefore, the introduction of split gaps concentrates the electric field within it, which is favorable for enhancing interaction between THz waves and the surrounding medium. Meanwhile, the high *Q*-factor and the strong localized electromagnetic fields in the split gaps provide a platform for enhanced interaction between the graphene and electric field inside the gaps, which has better amplitude modulation capacity than dielectric bars without split gaps.

**Figure 3:**
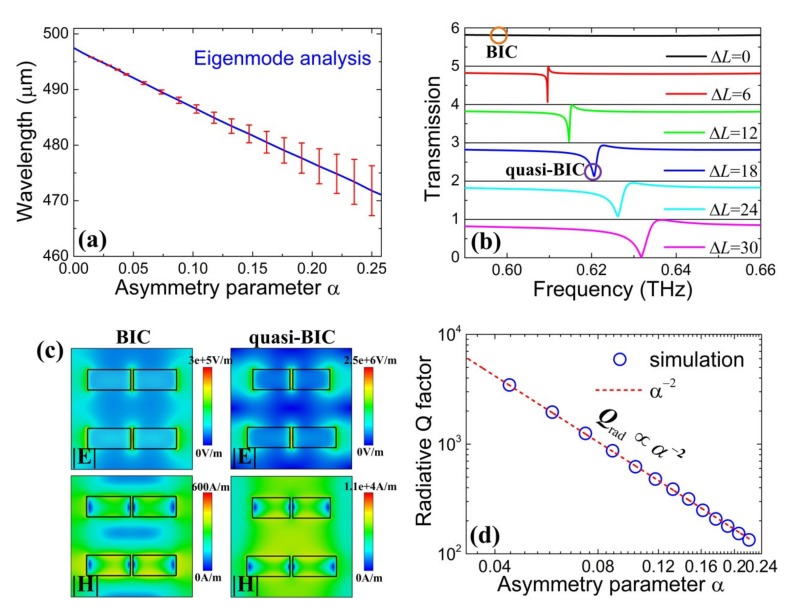
(**a**) Eigenmodes spectra with incident wavelength and asymmetry parameter *α*. Error bars show the magnitude of the mode inverse radiation lifetime. (**b**) Transmission spectra with a different length difference Δ*L*. (**c**) Distribution of electric and magnetic field amplitude for bound states in the continuum (BIC) and quasi-BIC labeled (**b**). (**d**) Radiative *Q*-factor of quasi-BIC with asymmetry parameter *α*.

For symmetry-protected BIC, it has an infinite *Q*-factor and could be transformed into quasi-BIC by breaking the symmetry of the unit cell structure, leading to a sharp Fano feature response. With the mechanism of BIC and excitation of quasi-BIC, one can control the radiation damping rate and engineer *Q*-factor of resonance. Hence, it is important to investigate the physical mechanism of symmetry-protected BIC and quasi-BIC Fano resonance in the dielectric metasurface. For the convenience of analysis, the lossless and infinite structure is adopted and the eigenmodes spectra are calculated numerically using an eigenmode solver in the software. The eigenmodes of the metasurface are treated as self-standing electromagnetic excitations with the complex eigenfrequency *ῶ* = *ω*_1_ − *iω*_2_, where *ω*_1_ is the resonant frequency and *ω*_2_ is the damping rate. Usually, two terms including the radiation damping and the material loss damping contribute to the damping rate *ω*_2_. With the losslessness of the structure, the damping rate is only from the radiation damping and the inverse radiation lifetime can be calculated as a sum of radiation losses into all open radiation channels. Thus, the mode eigenfrequency and the inverse radiation lifetime can be obtained with a different asymmetry parameter *α.* In Figure 3a, with *α* = 0, the symmetry-protected BIC can be supported, which has zero error bar and the transmission spectrum cannot be observed. The BIC state is unstable against the perturbation with breaking in-plane symmetry, which induces leakage of BIC and thus leads to the quasi-BIC with the mode inverse radiation lifetime increasing (as the error bar increases). Moreover, the transmission spectra with a different length difference Δ*L* are simulated in Figure 3b. Clearly, for Δ*L* = 0 (i.e., *α* = 0), no transmission dip can be observed and the linewidth vanishes. With Δ*L* increasing, the transmission spectra show distinct Fano resonance and its linewidth increasing, which confirms the results of the eigenmodes analysis in Figure 3a. Meanwhile, the resonant frequency shifts toward higher frequency with Δ*L* increasing because the bar length shortening increases the excitation energy of the Fano resonance [19]. In addition, the distribution of electric and magnetic field amplitude for the BIC and the quasi-BIC within the unit cell are simulated in Figure 3c, where the BIC and the quasi-BIC are marked with circles in Figure 3b. Clearly, the electric fields are mostly confined at both ends and middle gaps of dielectric bars and the magnetic fields are confined at the inner top and bottom sides of each bar. Most importantly, one can find that BIC and quasi-BIC have similar electric and magnetic field distributions, which demonstrate the quasi-BIC resonance coming from the BIC and Fano resonance is BIC-inspired. In Figure 3d, the relationship between the radiative *Q*-factor and the asymmetry parameter *α* is investigated. The dependence of the radiative *Q*-factor of quasi-BIC on the asymmetry parameter meets the inverse quadratic law (*Q*_rad_ ∝ *α*^−2^), which is consistent with the results reported by Koshelev et al. [25] and also demonstrates this Fano resonance is BIC-inspired. Hence, the unit cell with breaking in-plane symmetry is necessary to obtain a sharp quasi-BIC Fano resonance where its position and linewidth can be adjusted by the asymmetry parameter.

**Figure 4:**
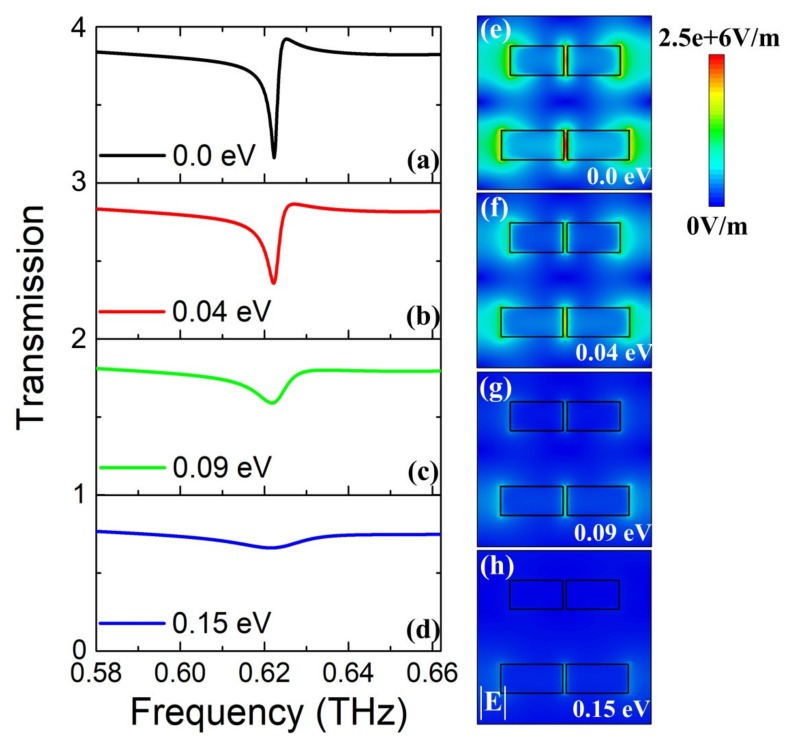
(**a**–**d**) Transmission spectra of the proposed metasurface structure by shifting the Fermi level of the graphene monolayer, where the temperature of STO is fixed as 300 K. (**e**–**h**) The corresponding electric field amplitude distributions at resonance frequencies shown in (**a**–**d**), respectively.

Most importantly, the tunable characteristic of the proposed metasurface structure is the main focus of our study. As we know, the modulation effect of graphene arises from the tunable conductivity by shifting its Fermi level *E_f_*. With the Fermi level *E_f_* increasing, the real part of conductivity increases remarkably, leading to the enhanced absorption and the modulation of resonance strength. In Figure 4, the transmission spectra and the corresponding electric field distributions are simulated to investigate the modulation and interaction of graphene monolayer with the quasi-BIC Fano resonance, where the temperature of STO is fixed as 300 K. When Fermi level *E_f_* is 0 eV, there is a sharp resonance dip in the transmission spectrum, as shown in Figure 4a. By increasing Fermi level *E_f_* from 0 eV to 0.15 eV, the transmission amplitude of the Fano resonance increases rapidly whereas resonance frequency is nearly unchanged, as shown in Figure 4a–d. With Fermi level *E_f_* increasing, the resonance strength undergoes a large change and nearly disappears up to 0.15 eV in Figure 4d, indicating the switch-off of Fano resonance. Moreover, the corresponding electric field distributions at resonances under different Fermi level *E_f_* are illustrated in Figure 4e–h, to reveal the interaction and coupling effect between graphene and the dielectric bars array. With Fermi level *E_f_* increasing, the electric field amplitude becomes weak, especially for the split gaps. This is because with Fermi level *E_f_* increasing, leading to more free carrier absorption of graphene and the suppression of resonance in the dielectric metasurface [46]. When Fermi level *E_f_* goes up to 0.15 eV, as shown in Figure 4h, the electric field amplitude nearly disappears, indicating the switch-off of resonance strength. Moreover, to quantify the change of the transmission amplitude, the modulation depth is employed to evaluate the modulation performance and defined as Δ*T* = (*T* − *T*_0_) × 100%, where *T* and *T*_0_ represent the transmission amplitude at resonance dip with different Fermi level *E_f_* and the reference Fermi level *E_f_* = 0 eV, respectively. By varying Fermi level, Δ*T* can be actively controlled and the largest Δ*T* can be achieved as 49.91% with Fermi level *E_f_* at 0.15 eV, which demonstrates the proposed structure can be utilized as an active THz modulator.

**Figure 5:**
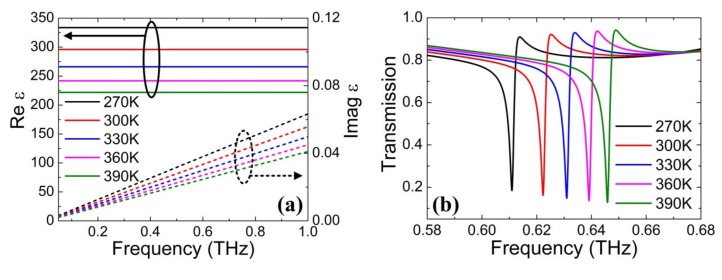
(**a**) Real and imaginary part of STO permittivity at different temperatures. (**b**) Transmission spectra of the proposed metasurface structure under different temperatures of the STO film, where the Fermi level of the graphene monolayer is fixed as 0 eV.

On the other hand, the tunability of resonance frequency is also studied by controlling the temperature of the STO film, whose permittivity is temperature-dependent. The permittivity of the STO material is modeled and calculated according to Equations (3)–(5). The calculated results are shown in Figure 5a, in which the real part of permittivity is plotted on the left axis, and the imaginary part is plotted on the right axis with temperature ranging from 270 K to 390 K. Clearly, the real part of permittivity increases largely from 221, 241, 266, and 295 to 333 with the temperature decreasing from 390 K to 270 K, due to the softening of the ferroelectric soft mode [44]. Meanwhile, the imaginary part of permittivity gradually decreases with increasing temperature and increases with an increasing frequency. These results indicate that the permittivity and refractive index of STO is temperature-dependent and the resonance of THz metasurfaces composed of STO can be modulated. Then, the transmission spectra of the proposed structure at different temperatures are investigated in Figure 5b. When the temperature decreases from 390 K to 270 K, the resonance frequency gradually redshifts from 0.6458 THz to 0.6108 THz, demonstrating the resonance frequency can be tuned actively. Besides, the resonance amplitude is nearly unchanged and has good amplitude stability in the transmission spectrum with different temperatures.

## 4. Conclusions

In summary, we have demonstrated a tunable THz dielectric metasurface constructed by split gap bars array, STO film, and graphene monolayer. With breaking in-plane symmetry and introducing split gaps in the unit cell, the quasi-BIC Fano resonance with high *Q*-factor and strongly localized electric fields in gaps can be obtained. Moreover, the eigenmodes analysis demonstrated the existence of symmetry-protected BIC and the radiative *Q*-factors exhibited the inverse square relation on the asymmetry parameter of the unit cell structure. For the first time to the best of our knowledge, it was found that both the amplitude and the resonance frequency of quasi-BIC Fano resonance can be tuned independently with controlling Fermi level of graphene and temperature of the STO film, respectively. The transmission amplitude can be modulated from 16.17% to 66.08% with Fermi level shifting from 0 eV to 0.15 eV and the resonance frequency can be tuned from 0.6108 THz to 0.6458 THz with the temperature of the STO film changing from 270 K to 390 K. Our structure opens a novel way for active control quasi-BIC Fano resonance metasurfaces that will design versatile devices such as modulators, filters, and biosensors.

## References

[1] Tonouchi M. (2007). Cutting-edge terahertz technology. Nat. Photonics.

[2] Ahmadivand A., Gerislioglu B., Ahuja R., Mishra Y.K. (2020). Terahertz plasmonics: The rise of toroidal metadevices towards immunobiosensings. Mater. Today.

[3] Pitchappa P., Kumar A., Prakash S., Jani H., Venkatesan T., Singh R. (2019). Chalcogenide phase change material for active terahertz photonics. Adv. Mater..

[4] Chen H.-T., Taylor A.J., Yu N. (2016). A review of metasurfaces: Physics and applications. Rep. Prog. Phys..

[5] Ahmadivand A., Gerislioglu B., Ramezani Z. (2019). Gated graphene island-enabled tunable charge transfer plasmon terahertz metamodulator. Nanoscale.

[6] Schurig D., Mock J.J., Justice B.J., Cummer S.A., Pendry J.B., Starr A.F., Smith D.R. (2006). Metamaterial electromagnetic cloak at microwave frequencies. Science.

[7] Luk’yanchuk B., Zheludev N.I., Maier S.A., Halas N.J., Nordlander P., Giessen H., Chong C.T. (2010). The Fano resonance in plasmonic nanostructures and metamaterials. Nat. Mater..

[8] West P.R., Ishii S., Naik G.V., Emani N.K., Shalaev V.M., Boltasseva A. (2010). Searching for better plasmonic materials. Laser Photonics Rev..

[9] Chu C.H., Tseng M.L., Chen J., Wu P.C., Chen Y.-H., Wang H.-C., Chen T.-Y., Hsieh W.T., Wu H.J., Sun G. (2016). Active dielectric metasurface based on phase-change medium. Laser Photonics Rev..

[10] Zhao Q., Zhou J., Zhang F., Lippens D. (2009). Mie resonance-based dielectric metamaterials. Mater. Today.

[11] He X., Lin F., Liu F., Shi W. (2020). Tunable strontium titanate terahertz all-dielectric metamaterials. J. Phys. D Appl. Phys..

[12] Yahiaoui R., Chung U.C., Elissalde C., Maglione M., Vigneras V., Mounaix P. (2012). Towards left-handed metamaterials using single-size dielectric resonators: The case of TiO_2_-disks at millimeter wavelengths. Appl. Phys. Lett..

[13] He X., Liu F., Lin F., Shi W. (2019). Investigation of terahertz all-dielectric metamaterials. Opt. Express.

[14] Zhang H., Zhang X., Xu Q., Tian C., Wang Q., Xu Y., Li Y., Gu J., Tian Z., Ouyang C. (2018). High-efficiency dielectric metasurfaces for polarization-dependent terahertz wavefront manipulation. Adv. Opt. Mater..

[15] Arbabi A., Horie Y., Bagheri M., Faraon A. (2015). Dielectric metasurfaces for complete control of phase and polarization with subwavelength spatial resolution and high transmission. Nat. Nanotechnol..

[16] Chen S., Chen Z., Liu J., Cheng J., Zhou Y., Xiao L., Chen K. (2019). Ultra-narrow band mid-infrared perfect absorber based on hybrid dielectric metasurface. Nanomaterials.

[17] Chen X., Fan W. (2019). Ultrahigh-*Q* toroidal dipole resonance in all-dielectric metamaterials for terahertz sensing. Opt. Lett..

[18] Tong W., Gong C., Liu X., Yuan S., Huang Q., Xia J., Wang Y. (2016). Enhanced third harmonic generation in a silicon metasurface using trapped mode. Opt. Express.

[19] Sun G., Yuan L., Zhang Y., Zhang X., Zhu Y. (2017). *Q*-factor enhancement of Fano resonance in all-dielectric metasurfaces by modulating meta-atom interactions. Sci. Rep..

[20] Limonov M.F., Rybin M.V., Poddubny A.N., Kivshar Y.S. (2017). Fano resonances in photonics. Nat. Photonics.

[21] Fan K., Shadrivov I.V., Padilla W.J. (2019). Dynamic bound states in the continuum. Optica.

[22] Monticone F., Alù A. (2017). Bound states within the radiation continuum in diffraction gratings and the role of leaky modes. New J. Phys..

[23] Friedrich H., Wintgen D. (1985). Interfering resonances and bound states in the continuum. Phys. Rev. A.

[24] Abujetas D.R., Van Hoof N., Ter Huurne S., Rivas J.G., Sánchez-Gil J.A. (2019). Spectral and temporal evidence of robust photonic bound states in the continuum on terahertz metasurfaces. Optica.

[25] Koshelev K., Lepeshov S., Liu M., Bogdanov A., Kivshar Y. (2018). Asymmetric metasurfaces with high-*Q* resonances governed by bound states in the continuum. Phys. Rev. Lett..

[26] Cong L., Singh R. (2019). Symmetry-Protected Dual Bound States in the Continuum in Metamaterials. Adv. Opt. Mater..

[27] Kodigala A., Lepetit T., Gu Q., Bahari B., Fainman Y., Kanté B. (2017). Lasing action from photonic bound states in continuum. Nature.

[28] Tittl A., Leitis A., Liu M., Yesilkoy F., Choi D.-Y., Neshev D.N., Kivshar Y.S., Altug H. (2018). Imaging-based molecular barcoding with pixelated dielectric metasurfaces. Science.

[29] Han S., Cong L., Srivastava Y.K., Qiang B., Rybin M.V., Kumar A., Jain R., Lim W.X., Achanta V.G., Prabhu S.S. (2019). All-Dielectric Active Terahertz Photonics Driven by Bound States in the Continuum. Adv. Mater..

[30] Savo S., Shrekenhamer D., Padilla W.J. (2014). Liquid crystal metamaterial absorber spatial light modulator for THz applications. Adv. Opt. Mater..

[31] Cheng J., Fan F., Chang S. (2019). Recent progress on graphene-functionalized metasurfaces for tunable phase and polarization control. Nanomaterials.

[32] Wang X., Meng H., Deng S., Lao C., Wei Z., Wang F., Tan C., Huang X. (2019). Hybrid Metal Graphene-Based Tunable Plasmon-Induced Transparency in Terahertz Metasurface. Nanomaterials.

[33] Jeong Y.G., Bahk Y.M., Kim D.S. (2019). Dynamic Terahertz Plasmonics Enabled by Phase-Change Materials. Adv. Opt. Mater..

[34] Chen X., Fan W., Song C. (2018). Multiple plasmonic resonance excitations on graphene metamaterials for ultrasensitive terahertz sensing. Carbon.

[35] Chen X., Fan W. (2017). Study of the interaction between graphene and planar terahertz metamaterial with toroidal dipolar resonance. Opt. Lett..

[36] He X., Lin F., Liu F., Zhang H. (2020). Investigation of Phonon Scattering on the Tunable Mechanisms of Terahertz Graphene Metamaterials. Nanomaterials.

[37] Guan S., Cheng J., Chen T., Chang S. (2019). Bi-functional polarization conversion in hybrid graphene-dielectric metasurfaces. Opt. Lett..

[38] Bakan G., Gerislioglu B., Dirisaglik F., Jurado Z., Sullivan L., Dana A., Lam C., Gokirmak A., Silva H. (2016). Extracting the temperature distribution on a phase-change memory cell during crystallization. J. Appl. Phys..

[39] Gerislioglu B., Bakan G., Ahuja R., Adam J., Mishra Y.K., Ahmadivand A. (2020). The Role of Ge2Sb2Te5 in Enhancing the Performance of Functional Plasmonic Devices. Mater. Today Phys..

[40] Yahiaoui R., Němec H., Kužel P., Kadlec F., Kadlec C., Mounaix P. (2011). Tunable THz metamaterials based on an array of paraelectric SrTiO3 rods. Appl. Phys. A.

[41] Němec H., Kužel P., Kadlec F., Kadlec C., Yahiaoui R., Mounaix P. (2009). Tunable terahertz metamaterials with negative permeability. Phys. Rev. B.

[42] Ospanova A.K., Karabchevsky A., Basharin A.A. (2018). Metamaterial engineered transparency due to the nullifying of multipole moments. Opt. Lett..

[43] Zhu H., Chen S., Wen J., Wang J., Chen L. (2019). Graphene-based metasurfaces for switching polarization states of anomalous reflection and focusing. Opt. Lett..

[44] Kužel P., Kadlec F. (2008). Tunable structures and modulators for THz light. C. R. Phys..

[45] Xiao S., Liu T., Zhou C., Jiang X., Cheng L., Liu Y., Li Z. (2019). Strong interaction between graphene and localized hot spots in all-dielectric metasurfaces. J. Phys. D Appl. Phys..

[46] Van Hoof N.J.J., Ter Huurne S.E.T., Vervuurt R.H.J., Bol A.A., Halpin A., Rivas J.G. (2019). Diffraction enhanced transparency in a hybrid gold-graphene THz metasurface. APL Photonics.

